# Development and Feasibility of a Kinect-Based Constraint-Induced Therapy Program in the Home Setting for Children With Unilateral Cerebral Palsy

**DOI:** 10.3389/fbioe.2021.755506

**Published:** 2021-10-26

**Authors:** Hao-Ling Chen, Szu-Yu Lin, Chun-Fu Yeh, Ren-Yu Chen, Hsien-Hui Tang, Shanq-Jang Ruan, Tien-Ni Wang

**Affiliations:** ^1^ Department of Physical Medicine and Rehabilitation, National Taiwan University Hospital, National Taiwan University, Taipei, Taiwan; ^2^ School of Occupational Therapy, College of Medicine, National Taiwan University, Taipei, Taiwan; ^3^ Department of Industrial and Commercial Design, National Taiwan University of Science and Technology, Taipei, Taiwan; ^4^ Department of Electronic and Computer Engineering, National Taiwan University of Science and Technology, Taipei, Taiwan

**Keywords:** cerebral palsy, constraint-induced therapy, children, upper limb, virtual reality

## Abstract

**Introduction:** Cerebral palsy (CP) is the leading cause of childhood-onset physical disability. Children with CP often have impaired upper limb (UL) function. Constraint-induced therapy (CIT) is one of the most effective UL interventions for children with unilateral CP. However, concerns about CIT for children have been repeatedly raised due to frustration caused by restraint of the child’s less-affected UL and lack of motivation for the intensive protocol. Virtual reality (VR), which can mitigate the disadvantages of CIT, potentially can be used as an alternative mediator for implementing CIT. Therefore, we developed a VR-based CIT program for children with CP using the Kinect system.

**Aims:** The feasibility of the Kinect-based CIT program was evaluated for children with unilateral CP using a two-phase study design.

**Materials and Methods:** In phase 1, ten children with unilateral CP were recruited. To confirm the achievement of the motor training goals, maximal UL joint angles were evaluated during gameplay. To evaluate children’s perceptions of the game, a questionnaire was used. In phase 2, eight children with unilateral CP were recruited and received an 8 weeks Kinect-based CIT intervention. Performance scores of the game and outcomes of the box and block test (BBT) were recorded weekly.

**Results:** In phase 1, results supported that the design of the program was CIT-specific and was motivational for children with unilateral CP. In phase 2, game performance and the BBT scores began showing stable improvements in the fifth week of intervention.

**Conclusion:** It suggested the Kinect-based CIT program was beneficial to the motor function of the affected UL for children with unilateral CP. According to the results of this feasibility study, larger and controlled effectiveness studies of the Kinect-based CIT program can be conducted to further improve its clinical utility.

**Clinical Trial Registration:** ClinicalTrials.gov, NCT02808195; Comparative effectiveness of a Kinect-based unilateral arm training system vs. CIT for children with CP

## Introduction

Cerebral palsy (CP) is the leading cause of childhood-onset physical disability, with a rate of 2–3 per 1,000 live births (Johnson, 2002). Children with CP often have impaired upper limb (UL) function due to weakness, spasticity and loss of selective muscle activation, all of which can further limit children’s participation in activities of daily living, education and play (Sakzewski et al., 2009). Constraint-induced therapy (CIT) has been suggested as one of the most effective UL interventions for children with unilateral CP. Despite the accumulated evidence supporting the positive effects of CIT, concerns for children have been repeatedly raised due to the increase in frustration over restraint of the child’s less-affected UL and the lack of motivation for the intensive protocol (Hart, 2005; Smania et al., 2009; Gilmore et al., 2010; Lin et al., 2011). As virtual reality (VR) can provide a playful restraint context and intensive repetition of task practice in highly motivating games, it can mitigate the limitations of CIT and potentially can be used as an alternative mediator for implementing CIT for children with unilateral CP. It is hoped that VR rehabilitation can improve the acceptability and feasibility of CIT.

CIT, which is based on the theories of cortical neural plasticity and motor learning (Taub et al., 1998; Deluca et al., 2006), has been shown to be an effective intervention for improving UL function in children with unilateral CP (Chen et al., 2014b; Sakzewski et al., 2014b). The two key ingredients of CIT are restraint of the less-affected UL and intensive structured training of the affected UL (Eliasson et al., 2014). Medium to strong effects of CIT on UL function in children with CP have been found, according to systematic review studies (Chen et al., 2014b; Sakzewski et al., 2014b; Fonseca Junior et al., 2017). When the International Classification of Functioning, Disability and Health levels model (ICF) is used to classify the outcomes of CIT, children with CP show improved grasp ability and reduced reaction and movement times at posttest for body structure and function levels (Cope et al., 2010; Chen et al., 2013). For activity level, most previous studies have shown that unimanual speed and dexterity, quality of movements, and amount of use of the affected UL increase in children with CP after CIT intervention (Sakzewski, 2012; Chen et al., 2013; Thompson et al., 2015). Moreover, bimanual performance in children with CP also improves (Klingels et al., 2013; Geerdink et al., 2015). For participation level, after CIT, the participation in daily life of children with CP improves, as measured by the Canadian Occupational Performance Measure and the Functional Independence Measure for Children (Chen et al., 2014b; Geerdink et al., 2015). Although the many positive effects of CIT are well supported, it still has some limitations. One is that restraining a child’s less-affected UL may cause discomfort and frustration during the intervention (Psychouli et al., 2010; Case-Smith et al., 2012; Mancini et al., 2013). Another is that the intensive training protocol may limit the motivation and compliance of the child and inevitably the labor intensity (Sakzewski et al., 2014a).

VR, which presents a computer simulation of the real world that allows the user to experience the simulation through a human-machine interface (Holden, 2005), has been widely explored as a training tool for motor rehabilitation in children with CP (Snider et al., 2010; Chen et al., 2014a; Choi et al., 2021). In most of the studies to date, motor rehabilitation with a VR system has been found to improve children’s motivation to participate in motor training programs (Harris and Reid, 2005; Tatla et al., 2013). For motor-related outcomes, Chen et al. (Chen et al., 2014a) reviewed studies of VR effects on UL function in children with CP and used the ICF model to classify the outcome variables. The results showed that, after VR intervention, a large effect was found for participation level, a small effect for activity, and a medium effect for body structure and function levels in children with CP. Moreover, a rehabilitation-specific VR system has been reported to be more effective than a commercial program (Fluet et al., 2010). One possible reason is that a rehabilitation-specific VR system can be matched to each child’s motor capacity and designed for the goals of motor training (Chen et al., 2014a). However, the cost of building a rehabilitation-specific VR system is much higher than that of a commercially available system (e.g., the Kinect system). Decreasing the cost of the rehabilitation-specific VR system such that it is affordable for families with children with CP should be considered.

The benefits derived from the VR system include: 1) intensive repetition of task practice; 2) immediate visual and/or auditory feedback on performance; 3) highly motivating games as the mediator of motor training; 4) flexibility to individualize treatment parameters; and 5) performance of tasks that cannot be performed safely in a real-world environment (Levac et al., 2012; Zoccolillo et al., 2015). Most of the components of VR are essential for motor learning, which makes VR a potentially viable approach for integration into CIT intervention, an intervention based on motor learning (Grordon and Okita, 2010; Rostami et al., 2012). The benefits of VR also help mitigate the limitations of CIT, namely children’s frustration and decreased motivation. Rostami et al. (Rostami et al., 2012) combined VR and a CIT program for training UL function in children with unilateral CP. However, the VR game used in their study included bimanual tasks (e.g., a driving game). Unimanual training activities were suggested, according to CIT protocol (Gordon et al., 2005). Moreover, a volar resting splint was used as a restraint in their intervention; such a splint may negatively impact the child’s emotional and psychological well-being. Recently, Wii, a commercial VR system, has been integrated into CIT intervention. Some psychosocial benefits in terms of decreased parental stress and increased child engagement were found. However, as Wii games were not designed for therapeutic use, it might not provide appropriate grading methods for the rehabilitative goal (Wang et al., 2021). The Kinect camera is a low-cost and portable markerless motion capture sensor. Incorporating the Kinect with the Windows software development kit (SDK) allows users to create interfaces and games to achieve the goals of motor training (Mintal et al., 2015). The Kinect system has been successfully used to develop rehabilitation-specific programs for patients with Parkinson’s disease and stroke (Pastor et al., 2012; Palacios-Navarro et al., 2015). In view of this, the Kinect system may be applicable to the design of a CIT program for children with unilateral CP. Combination of the benefits of CIT and VR rehabilitation may help improve their clinical feasibility. To the best of our knowledge, no VR-based rehabilitation program was designed based on CIT principles using low-cost system.

Therefore, the purpose of the study was to develop a VR-based CIT program for children with unilateral CP using the Kinect system. Moreover, the feasibility of the Kinect-based CIT program was evaluated for children with unilateral CP using a two-phase study design. A feasibility study with a small sample size is designed for the partial foundation of future RCT research (Tickle-Degnen, 2013). Therefore, in the phase 1 study, the achievement of the CIT-specific design and the safety and motivation of the game were verified using motion analysis and a questionnaire on the children’s experience during 20 min of gameplay. The a priori hypothesis was that this motor rehabilitation program was CIT-specific, safe and motivational for children with unilateral CP. In the phase 2 study, the aim was to investigate the preliminary effects of Kinect-based CIT on motor function of the affected UL in children with unilateral CP. The a priori hypothesis was that the children with unilateral CP would have stably significant motor improvement, as evaluated by the performance score and the Box and Block test (BBT) after 18 h of Kinect-based CIT.

## Materials and Methods

### The “Adventure Island” Game

To develop our VR-based CIT program, Kinect for Windows SDK was used to provide an Application Programmer’s Interface (API) to the Kinect 2 sensor. The Kinect 2 sensor includes an RGB camera and an infrared emitter with an infrared depth sensor, which allows measurement of the movements of players in three dimensions and provides real-time feedback on movements while the game is played. The Kinect 2 API’s skeletal tracking functions provide position estimates for 25 anatomical landmarks on the head, neck, trunk, shoulders, elbows, hands, thumbs, hips, knees, ankles and feet with a frequency of 30 Hz. The game was developed using Unity, with computer graphics created in 3ds Max for three-dimensional (3D) animation and Photoshop for the user interface.

**FIGURE 1:**
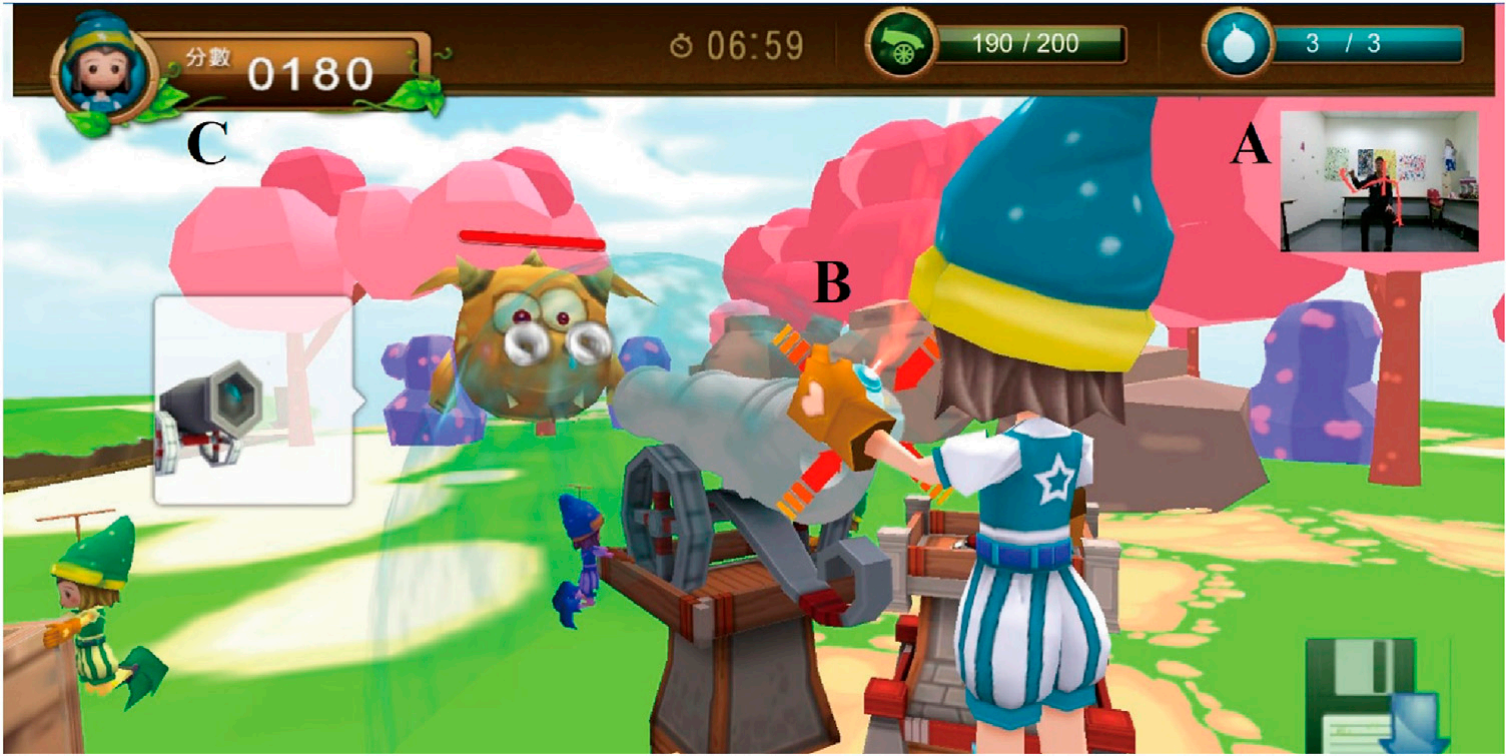
The game screen and the feedback during the gameplay. **(A)** A participant playing our Kinect game with a Kinect skeleton tracker. **(B)** The visual feedback on the upper limb movements of the player (Knowledge of performance). **(C)** The performance score during gameplay as another form of feedback (Knowledge of results).

This game was designed by a research team of occupational therapists, engineers and designers. The theme of this game was based on a feasibility survey of ten children with CP to determine the preferred game type prior to the game design. Based on the children’s favorite type of game, a game called “Adventure Island” was developed. In this game, a child acts as a warrior to defeat all the monsters and maintain peace on the island. The missions of the warrior are to collect cannonballs and then place the cannonballs in a fort to defeat the monsters (Figure 1).

**FIGURE 2:**
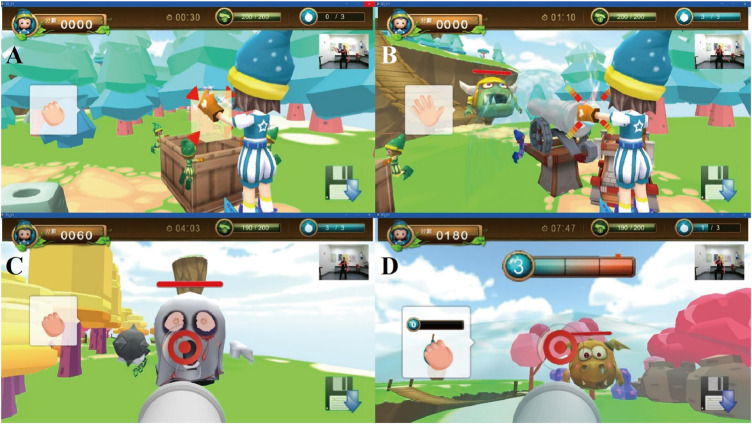
A Kinect-based CIT program was designed with three levels of task difficulty. In the preparation stage **(A)** and the first level **(B)**, the movement training goals include reaching, grasping, and releasing. In the second level **(C)**, the training goals include reaching, grasping, releasing, and aiming. In the third level **(D)**, the goals consist of reaching, grasping, releasing, aiming, and holding.

This game was developed for the UL rehabilitation of children with unilateral CP. Previous studies revealed that children with CP have difficulty in shoulder flexion, elbow extension, forearm supination, and voluntary movement of the fingers of the affected side (Bhardwaj and Sabapathy, 2011). In accordance with CIT protocol, the categories of arm-reaching, manipulation, and arm-hand tasks were addressed (Gordon et al., 2005; Abd El-Kafy et al., 2014). Therefore, the motor training goals of “Adventure Island” were to train the following movements of the affected UL: reaching, grasping, releasing, holding and aiming (Figure 2). In the game, before defeating the monsters, the player has to reach for and grasp the cannonballs. After collecting sufficient cannonballs, the player has to hold and/or aim the cannon to attack the monsters. Moreover, a pause mechanism during gameplay prevents inadequate compensatory movements (e.g., trunk flexion instead of upper limb movement) and ensures the player’s safety. The Kinect system can detect abnormal postures and movement patterns of the player during gameplay. If the player exhibits compensatory movement of the trunk, the game pauses and a warning appears on the screen to instruct the user to assume the correct position.

Two key components of CIT are restraint of the less-affected UL and intensive structured training of the affected UL. A contextual restraint is used instead of a physical restraint. The less-affected limb is gently restrained (i.e., the less-affected hand is placed on one knee) to accomplish in-game actions such as placing a hand on a box for support or pressing a button on a shield to defend against attacks by monsters (Figure 1). If the player uses the less-affected hand to play the game, the game pauses and a pop-up warning appears on the screen to instruct the player to put the less-affected hand on a knee.

To incorporate the concept of intensive structured training of the affected UL required for CIT, five strategies from motor learning theory were applied. First, the intensity of training is achieved by repetitive practice of the tasks in the game. For example, before defeating the monsters, the children have to collect cannonballs by repeating the tasks of reaching, grasping and releasing. Second, visual and auditory feedback are incorporated into the game design to encourage the children to participate (Figure 1). Knowledge of results (KR, information about the outcome of performing an action) and knowledge of performance (KP, information about the movement characteristics leading to the performance outcome) are two categories of the feedback. Both KR and KP are valuable for facilitating motor learning (Chen et al., 2016) and thus were adopted in our game design. In the game, the score indicating the defeated monsters is KR, while the performance of grasping cannonballs is KP. Since too much feedback may interfere with motor learning and retention (Hemayattalab and Rostami, 2010), 50% feedback (providing feedback for half of the trials) was chosen for the game. Third, a shaping skill was used for game design. It emphasizes progressively increasing the task difficulties in temporal and spatial dimensions, as well as accuracy of movement (Abd El-Kafy et al., 2014). During the cannonball gathering stage, cannonballs randomly appear in four directions relative to the player: inward, outward, upward and downward. There are three levels of game difficulty. At the game with higher difficulties, the movements are more complex and more challenging in temporal, spatial and accuracy (Figure 2). For example, in level 1, the player defeats the monster by putting cannonballs into the fort. When the child’s ability increase, the difficulty in spatial is increased in terms of moving the positions of the cannonballs more far away from the warrior. In level 3, the player must aim at the monster and hold that position for 3 s to inflict the greatest amount of damage to the monster. The temporal, spatial and accuracy difficulties of the required movements are higher than those of level 1. Moreover, to ensure smooth game progress and to improve the child’s sense of accomplishment, if the Kinect system cannot detect movement during the required time due to the child’s poor motor capacity, the task is automatically accomplished by the system. Fourth, personalized game difficulty was adopted to provide just the right amount of challenge for each child with CP according to his/her motor ability. At the beginning of the game, the maximal reaching distances of both hands are measured by the Kinect system. The children with CP are guided by bubbles to reach in five different directions, namely, outward, inward, upward, downward and forward, for 15 s in each direction. When the child reaches the bubble, it moves farther and farther away to induce the child’s maximal reaching distance without trunk movement. After the evaluation, the personalized game difficulty is decided. Fifth, user interfaces for both the therapists and the children were designed. In the therapist’s interface, a therapist can choose the difficulty of the game level to fit the child’s ability. In addition, the system outputs data on the child’s performance for the therapist to understand the progress of the intervention. For the children’s interface, a leaderboard is provided so that the children can see their own performances and compare them with those of other children playing the game, adding an element of competition that might help to inspire them. Moreover, a weapon collection book is provided to enhance the motivation to play the game. Upon finishing the game, child is awarded a new style of cannonball, and this cannonball appears in the subsequent gameplay session.

### Phase 1

#### Participants

Ten 5- to 12 year-old children with unilateral CP were recruited for this phase, and a caregiver (e.g., a parent) provided informed consent. The inclusion criteria were 1) diagnosis of CP with one or more affected side; 2) considerable nonuse of the affected upper limb (amount-of-use score of the Pediatric Motor Activity Log <2.5); 3) no excessive muscle tone (Modified Ashworth Scale ≤2 at any joint of the upper limb); and 4) no severe cognitive, visual, or auditory disorders according to medical documents, parental reports, and the examiner’s clinical observation.

#### Procedure

The study of this phase was conducted in a motion laboratory to ensure safety. The game was run on a laptop running Windows 8, to which the Kinect 2 sensor was connected. The game was displayed on a 1920*1,080 resolution Acer projector placed 2 m distant from the participants. Participants played the game for approximately 20 min. The game was presented with increasing difficulty. An experienced occupational therapist (SYLin) was present during gameplay to ensure the safety of the participants and to prevent any possible adverse events. After playing the game, the participants were asked to complete a questionnaire on their gameplay experiences.

#### Evaluation

To confirm the achievement of the motor training goals of CIT, a 6-camera motion analysis system (Vicon MX13+, Oxford Metrics Group, United Kingdom) was used to record marker trajectory data during gameplay. Eleven infrared retroreflective markers with a diameter of 14 mm were attached to the bony landmarks, including the thorax (Spinous process of the seventh cervical vertebra, spinal process of the eighth thoracic vertebra, and deepest point of Incisura Jugularis), and to the affected UL at the humerus (glenohumeral rotation center, lateral and medial epicondyle), forearm (radial and ulnar styloid), and hand (second metacarpal bone base, second and fifth metacarpophalangeal joint). Trunk and UL movement during four cannonball-collecting sessions and three monster attack sessions of gameplay were recorded. Each session was recorded for 1 minute. Maximal joint angles of the shoulder, elbow and wrist during these sessions were analyzed. These data were used in the evaluation of whether multi-directional movements of multiple joints of the UL could match the training goals for intensive motor training. Moreover, the grasping actions were recorded by our Kinect program by detecting changes in the distance from the hand to the thumb landmarks of the Kinect skeleton. The recorded grasping actions were used for evaluating whether Kinect-CIT could provide intensive training of the affected UL.

To evaluate the children’s perception of playing the game and achievement of the CIT-specific design, a questionnaire was designed by our research team. Some of the questions of the questionnaire focused on five attributes of game concepts: enjoyability, safety, challenge, acceptability, and skill at the game (Hanna et al., 2004). The other questions focused on comparing the children’s experience of their conventional rehabilitation and this Kinect-based CIT program. The Smileyometer rating scale combined with a visual analogue scale, which is frequently used to evaluate children’s experiences with technology, was used in our questionnaire (Sim and Cassidy, 2013). As shown in Appendix A of Supplementary Material, each item of this questionnaire was scored on a 10 cm-long horizontal line with six faces showing a range of emotions from happy to sad above it. The participant was instructed to mark the line to indicate the level of agreement. The total score for each item ranged from 0 to 10, based on the distance in millimeters from the right end of the line. A longer distance represented more positive conditions. Items 5.1 and 5.2 of the questionnaire, which were on level of fatigue, were reverse scored. Part B of the questionnaire also had a short interview focused on comparing the children’s experiences of and feelings about their conventional rehabilitation and this Kinect-based CIT program.

### Phase 2

#### Participants

This phase was a preliminary study and was part of a clinical trial (Trial Registration: ClinicalTrials.gov NCT02808195; Comparative effectiveness of a Kinect-based unilateral arm training system vs CIT for children with CP). In this clinical trial, participants were randomly assigned either to a Kinect-based CIT or a CIT group with a computerized web-based randomization service (http://www.randomization.com/). Eight 5- to 12 year-old children with unilateral CP were recruited and informed consent was obtained from caregivers. The inclusion criteria were the same as those in the Phase 1 study.

#### Procedure and Outcome Measures

The Kinect-based CIT program consisted of 18 h of training sessions over a period of 8 weeks. This training program was performed at the participants home and supervised by an occupational therapist (SYLin). At the beginning, the OT prompted the participant to perform each movement to the best of his or her ability and modified the parameters of the game. The OT also monitored each session of intervention for possible adverse events. For measurement of the intervention effects on motor function of the affected UL, the performance score on the game during the intervention and a clinical assessment with the box and block test (BBT) after the intervention were recorded weekly.

##### Performance Score

Performance scores, calculated by dividing raw scores by total scores, were recorded by the Kinect system. Before starting the game, the therapist adjusted the game difficulty based on the motor capacity of each participant. Harder difficulty in the game would lead to a higher maximal total score. The raw score was calculated according to the success rate in grasp actions and the total gameplay completion time. The performance score corresponded to the motor function of the affected UL in children with unilateral CP and was used for evaluating the effectiveness of the intervention.

##### The Box and Block Test (BBT)

The box and block test (BBT) is a standard assessment for evaluating manual function. The BBT was used for measuring the effectiveness of the intervention on the gross manual dexterity of the affected UL in the children with unilateral CP. The box was divided into two equally square compartments by a vertical board. The individuals were asked to grasp and transport a one-inch block from one compartment to the other, and the number of blocks transported successfully in 60 s was recorded as the clinical outcome for gross manual dexterity of the upper extremity. To reduce the practice effect, a 15 s practice trial was performed before the actual assessment started (Jongbloed-Pereboom et al., 2013a)*.* A previous study has reported that the reliability and responsiveness of the BBT in measuring the improvement of motor function after interventions for children with CP are acceptable (Araneda et al., 2019).

#### Data Analysis

Descriptive statistics were used to summarize the demographic characteristics of the participants and the motor performances in eight weekly interventions. Performance scores and outcomes of the BBT were recorded weekly after each intervention. These repeated measurements were obtained at the first week as the baseline and at the other 7 weeks, and they were correlated within participants. Thus, the Generalized Estimating Equation (GEE), a widely-used, robust method for cross-sectional data, was used to handle the dependence in the data and to measure the effectiveness on motor function of the affected UL across 8 weeks in the small sample (Shih, 1997; Zorn, 2001; Hanley et al., 2003). The first-order autoregressive (AR1) correlation structure was considered for the GEE linear model, adjusting for the level of MACS (Manual Ability Classification System) of the participants. The selection of all models was based on the smallest independence model criterion (QIC). All statistical analyses were conducted in SPSS version 20, and all the levels of significance were α= 0.05.

## Results

### Phase 1

The demographic data of the recruited participants are listed in Table 1A. No adverse events occurred during the gameplay. During the 20 min gameplay sessions, the children performed a mean of 72 grasping actions (range 54–108 repetitions). Two children completed all three levels of the game in 20 min. Six children finished the first 2 levels. Two children completed only the first level.

**TABLE 1A:** Demographic data of participants in feasibility study (A) and effectiveness study (B).

Subject	Age (years)	Sex	Affected side	MACS level
1	8.33	M	L	2
2	11.67	F	R	2
3	5.75	M	R	2
4	11.83	M	R	1
5	8	M	L	1
6	9.58	M	R	1
7	11.5	M	L	3
8	7.33	M	R	2
9	9.83	F	L	1
10	9	F	L	2

**TABLE 1B:** 

Subject	Age (years)	Sex	Affected side	MACS level
1	5.50	F	R	1
2	8.42	F	L	2
3	11.00	M	L	1
4	8.25	M	R	2
5	6.75	F	R	3
6	5.50	M	R	2
7	6.42	F	R	2
8	6.58	F	R	1

M, Male; F, Female; R, Right; L, Left.

Range of motion (ROM) of shoulder flexion, abduction, adduction, and elbow flexion, as well as wrist flexion and extension, are reported in Table 2A. Compensatory movements (i.e., trunk flexion) were also recorded (Table 2A). According to the results of the questionnaire, all of the children reported a positive experience (Table 2B), and all felt safe during the gameplay. Most of the children preferred the Kinect-based upper limb motor training system (75%) over the regular intervention (25%).

**TABLE 2A:** (A) Range of motion of upper limb joints and trunk during 20 min gameplay (B) Questions and results of the gameplay questionnaire.

Joint angles	Maximal value	Minimal value
shoulder abduction/adduction	96.95 (SD: 23.63)	−33.06 (SD: 50.63)
shoulder flexion	91.67 (SD:14.07)	11.90 (SD:8.31)
elbow flexion	91.92 (SD:15.94)	11.25 (SD:12.81)
wrist flexion	33.67 (SD:13.90)	−30.81 (SD:27.66)
trunk flexion/extension	33.37 (SD: 19.28)	−18.89 (SD:9.57)

**TABLE 2B:** 

Items		Score (higher score indicates more positive conditions)
Did you feel happy when playing this game?		8.62 (SD: 1.50)
Did you love this game?		8.27 (SD: 1.58)
Did you feel you were good at this game?		7.25 (SD: 1.88)
Was this game difficult for you?		6.99 (SD: 2.73)
Did you feel tired while playing the game?	Affected UL	4.84 (SD: 3.15)
	Less-affected UL	7.89 (SD: 2.87)
Do you want to play this game again?		8.13 (SD: 1.80)
Do you want to play this game at home?		7.00 (SD: 2.85)

### Phase 2

**FIGURE 3:**
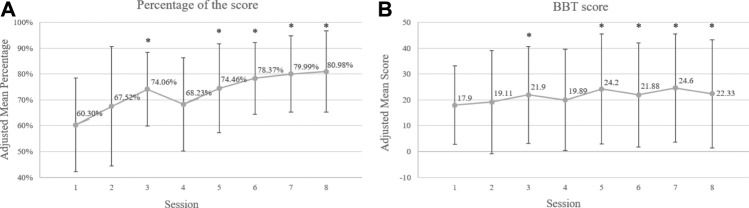
**(A)** Eight weeks of performance scores of the Kinect game and **(B)** outcomes of the BBT among children with unilateral CP over training sessions. * statistical significance of *p* ≤ 0.05 compared to the baseline.

The demographic data of the recruited participants are listed in Table 1B. For the performance in the game, the mean performance score at baseline was 60.30% (SD = 18.17%). The performance score increased significantly at the third (*p* = 0.002) and fifth (*p* = 0.002) weeks from baseline, and progression was stable from the fifth week to the last (week 6: *p* = 0.042; week 7: *p* = 0.001; week 8: *p* = 0.001). For the BBT, the mean score at baseline was 22.33 blocks (SD = 20.93). The mean score of the BBT increased significantly at the third (*p* = 0.001) and the fifth (*p* = 0.004) weeks from baseline, and progression was stable from the fifth week to the last, which was consistent with the performance scores (week 6: *p* = 0.019; week 7: *p* = 0.003; week 8: *p* = 0.005) (Figure 3) ***.***

## Discussion

This study aimed to develop a Kinect-based CIT program for children with unilateral CP. To our knowledge, this is the first VR program to be designed based on the concepts of CIT. Based on the results of the Phase 1 study, the program was successfully designed as a CIT-specific game for children with unilateral CP. Most of the children felt that Kinect-based rehabilitation was more interesting than conventional therapy, and they wanted to play the game again. Moreover, results of the Phase 2 study supported that our Kinect-based CIT program was effective in improving gross manual dexterity in the affected UL in children with unilateral CP.

### Development of a Kinect-Based CIT Program for Children With Unilateral CP

Restraint of the less-affected UL without negative emotion was successfully achieved in our program. The Kinect, a motion capture sensor, allows the design of a limb-specific game wherein the player must play the game using specific movements of the limb specified by the game designer. In our program, children with unilateral CP were asked to play the game using only their affected UL. Moreover, to prevent negative emotion resulting from the restraint, contextual restraint methods were adopted. In our program, when the children with CP put their less-affected hand on one knee, the box is held stable during bomb collection or the shield is raised to defend against attacks by monsters. According to the results of the questionnaire, all of the children enjoyed the gameplay and thought highly of it (>8). We found that during the gameplay, all of the children were involved in the context of the game and actively used their affected UL to play it. No children complained about the game requirements or refused to use their affected ULs to play the game.

Intensive training of the affected UL, one of the goals of CIT, was also achieved by our program. Multi-directional reaching and grasping were the two motor training tasks in our program. In view of this, we wanted to ensure that this game could induce children to perform the correct movements instead of compensatory movements. Recently, many commercially-available VR systems have been broadly used for rehabilitation in children with CP. However, some limits of the system itself may reduce the sensitivity of detection. For example, the Wii console only detects the acceleration of the remotes, so children can accomplish tasks by performing compensatory movements (Pastor et al., 2012). Thus, a Wii-based game might not be able to train ideal movements because the children only need to perform wrist or finger movements to accomplish all the tasks. A VR-based game using the Kinect system can eliminate this disadvantage. Based on the motion analysis data in Table 2A, our Kinect-based program induced active ROM of all UL joints. This result was in line with our training goal, namely, that the children should reach for and grasp cannonballs in multiple directions. In addition, the maximal angles of trunk flexion in the children with CP were greater than 30°. Trunk movement is a common compensatory movement during forward reach in children with CP (Jaspers et al., 2011). Thus, it is necessary to monitor trunk movement to prevent compensatory movement during gameplay, and a corresponding pausing mechanism triggered by excessive trunk movement is indispensable for postural correction. Moreover, according to the Kinect-based program, the children with unilateral CP could achieve an average of 72 repetitive grasps within 20 min of gameplay. This intensive amount of training is difficult to achieve in conventional training. Since intensive training is crucial to causing neural reorganization in the brain and improving the learning of motor and functional skills (Pereira et al., 2014), our Kinect-based CIT program can provide the opportunity for intensive and repetitive practice.

Strategies from motor learning theory, including the structured training required for CIT, were adopted, making the Kinect-based CIT program motivational for children with unilateral CP. One primary consideration in the game design was the pace of the game. In general, the speed requirements of commercial games are too fast for children with CP, and they may feel frustration and anxiety during gameplay. Moreover, a game with excessively fast movements may induce abnormal muscle tone in the affected UL during gameplay (Scholtes et al., 2006). To overcome the disadvantages of commercial games, our Kinect-based CIT game was designed based on the motor abilities of children with unilateral CP, and all levels of the game were graded by the difficulty of the motor requirements. All of the children responded that the most difficult part of the game was defeating the third monster, which required the children to perform aiming and holding tasks. This result supported our game setting of level of difficulty; the third level presented the greatest challenge. In addition, according to the results of the questionnaire, questions such as “Was this game difficult to you?” and “Did you feel you were good at this game?” suggested that the difficulty of the game may be moderate for children with CP. Our Kinect-based CIT program can provide just the right amount of challenge for children with unilateral CP.

### Improvement of Motor Function in Affected Upper Limb After Kinect-Based CIT Training

To evaluate the feasibility of our Kinect-based CIT program, a feasibility study for evaluating the potential effect in our program (i.e. Phase 2 study) was also performed. Based on the results of the performance scores recorded by the Kinect system and the outcomes of the BBT, both performances increased stably after 5 weeks of intervention, revealing the potential contribution to the affected UL motor function for the children with unilateral CP. However, the results showed decreases in the performance score and the outcomes of the BBT from the third week to the fourth week. For the performance score in our research, the positive impact of motor function was indicated in the third week (*p* = 0.002). Therefore, the therapist adjusted the game difficulty in the fourth week. The participants faced a new challenge and had not yet adapted, which might have caused the decline in the raw scores of participants in the game. In addition, a similar drop in BBT outcome could have been caused by motor reorganization in response to an unadaptable and challenging task. At the time of reorganization, the participants may have tried to integrate their skills to improve the quality of movement, which would have led to an increase in variability and more errors in motor performance (Thibaut and Toussaint, 2010; Jongbloed-Pereboom et al., 2013b). This decrease in motor performance during reorganization processes has also been found in the development of motor planning skills and handwriting (Meulenbroek and van Galen, 1988; Jongbloed-Pereboom et al., 2013b). Limited to our preliminary findings, however, more robust studies were needed to establish the effectiveness of the Kinect-based CIT training for children with unilateral CP.

### Clinical Implications

Regarding the clinical implications for intervention in the future, our findings reveal the potential benefits of using this Kinect-based CIT program for telerehabilitation for children with unilateral CP. The use of telerehabilitation, or using telecommunication devices to provide treatments and evaluations for clients, has been increasing recently (Peretti et al., 2017). One advantage of telerehabilitation is that medical services can be provided anywhere and anytime, and even directly in the client’s own home. It is individualized, effective and flexible, and it might reduce the risk of spreading diseases (e.g., COVID-19) and other infections (Anton et al., 2018; Molinaro et al., 2020; da Silva et al., 2021). Several studies have reported that game-based telerehabilitation tends to contribute to the improvement of UL motor function in children with CP (Golomb et al., 2010; da Silva et al., 2021). The Kinect system is an inexpensive and widely-used sensor for treatment, and the therapist can refer to the Kinect records to give advice on the movements and levels of game difficulty to clients and their parents periodically. Thus, future studies could evaluate the effectiveness of this Kinect-based CIT program delivered through telerehabilitation on motor function of the affected UL in children with unilateral CP.

### Study Limitations

This study had some limitations. First, the sample size was small, and relatively high-functioning participants were recruited for our feasibility study. The results of this study thus might not be generalizable to other types of CP. Second, limited to the present preliminary study to evaluate the potential effects of our VR-based CIT program, further larger and controlled studies are needed to confirm the more robust effectiveness of our Kinect-based CIT program, which may help to improve the clinical utility of the Kinect-based CIT program.

## Conclusion

A Kinect-based CIT program for children with unilateral CP has been developed. The feasibility study indicated that this motor rehabilitation program was motivational and CIT-specific. Our Kinect-based CIT program successfully mitigated the disadvantages of CIT, which may help improve the acceptability and feasibility of CIT for children with unilateral CP. Moreover, our Kinect-based CIT program can improve motor function in the affected UL. Larger and controlled effectiveness studies of the Kinect-based CIT program should be conducted in the future to improve its clinical utility.

## Data Availability

The raw data supporting the conclusions of this article will be made available by the authors, without undue reservation.

## Abbreviations

CP, Cerebral palsy; UL, Upper limb; CIT, Constraint-induced therapy; VR, Virtual reality; SDK, Software development kit; API, Application Programmer’s Interface; 3D, three-dimension; KR, Knowledge of results; KP, Knowledge of performance; ROM, Range of motion; MACS, Manual Ability Classification System; BBT, the box and block test; SD, Standard Deviation.
