# Supplementary material for: Development and Feasibility of a Kinect-Based Constraint-Induced Therapy Program in the Home Setting for Children With Unilateral Cerebral Palsy
Source: Front Bioeng Biotechnol. 2021 Oct 26;9:755506. doi: 10.3389/fbioe.2021.755506 (PMC8576521; doi:10.3389/fbioe.2021.755506)
Supplement: Supplementary file 1 [file Presentation1.PDF]

Supplementary Material

1 Appendix A: The gameplay questionnaire

**Part A. The Smileyometer rating questionnaire**	
Items	Score 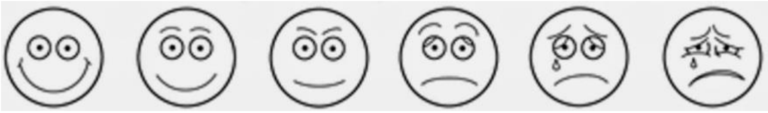
1. Did you feel happy when playing this game?	Happy —— Unhappy
2. Did you love this game?	Like —— Dislike
3. Did you feel you were good at this game?	Good —— Not good
4. Was this game difficult for you?	Easy —— Difficult
5.1. Did you feel tired while playing the game? (***Right*** upper extremity)	Tired —— Not tired
5.2. Did you feel tired while playing the game? (***Left*** upper extremity)	Tired —— Not tired

6. Do you want to play this game again?	Want ———————— Do not want
7. Do you want to play this game at home?	Want ———————— Do not want
**Part B. Interview**	
8. What is the feeling when you are playing this game?	Exciting Funny Interesting Beatiful Ugly<br>Childlike Surprise Boring Excellent Dull Confused
9. Are you feel safe when you are playing this game? (e.g., afraid of falling down the chair)	
10. What is the most difficult task in this game?	
11. Do you have any questions or anything you are not understood during gameplay?	
12. Do you have any rehabilitative treatments usually? What's the training of rehabilitation you do?	
13. Which rehabilitative tasks are more interesting to you? Why?	☐ The Kinect-based upper limb motor training system<br>☐ The regular rehabilitative training<br>Reason:

14. Do you prefer to do the training of the Kinect program or regular rehabilitation? Why?	☐ The Kinect-based upper limb motor training system<br><br>☐ The regular rehabilitative training<br><br>Reason:
